# Supplementary material for: Identification of photoperiod-induced specific miRNAs in the adrenal glands of Sunite sheep (Ovis aries)
Source: Front Vet Sci. 2022 Jul 22;9:888207. doi: 10.3389/fvets.2022.888207 (PMC9354845; doi:10.3389/fvets.2022.888207)
Supplement: Supplementary file 1 [file Data_Sheet_1.ZIP › Supplementary file/miRDeep2 file.zip!/miRDeep2 file/Know_Structure/LP42A1/oar-mir-487a.pdf]

miRBase precursor : oar-mir-487a
Total read count : 79
oar-miR-487a-5p read count : 22
oar-miR-487a-3p read count : 56
remaining reads : 1

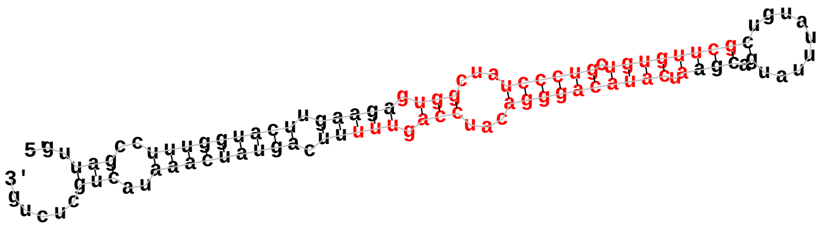

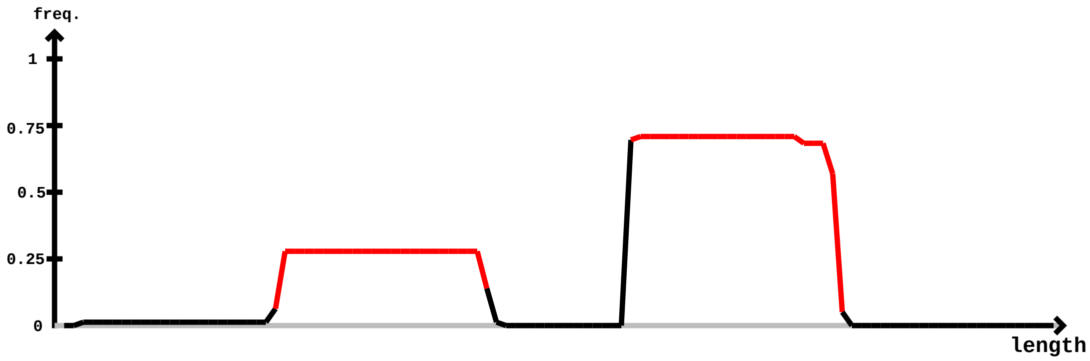


```
                                                                      oar-miR-487a-3p
                          oar-miR-487a-5p
5'- guuagccuuugguacuugaagaguggcuaucccugcuguguucgcuguauuuaugacgaaucauacagggacauccaguuuuucaguaucaaauacugcucug   -3'    exp
    ..(((..(((((((((.(((((.(((...((((((.(((((((((.........).)))).))))))))))...))).))))).)))))))))..))).....    reads      mm         sample
    .uuagccuuugguacuugaag..................................................................................    1          0          seq
    .....................aguggcuaucccugcuguguuc............................................................    4          0          seq
    .....................aguggcuaucccugcuguguucg...........................................................    1          0          seq
    ......................guggcuaucccugcuguguuc............................................................    7          0          seq
    ......................guggcuaucccugcuguguucg...........................................................    9          0          seq
    ......................guggcuaucccugcuguguucgc..........................................................    1          0          seq
    ..........................................................aaucauacagggacaucc...........................    2          0          seq
    ..........................................................aaucauacagggacauccagu........................    9          0          seq
    ..........................................................aaucauacagggacauccaguu.......................    41         0          seq
    ..........................................................aaucauacagggacauccaguuu......................    3          0          seq
    ...........................................................aucauacagggacauccaguuu......................    1          0          seq
```
